# *In vivo* effects of javamide-I/-II on metabolic, hepatic, cardiovascular and inflammatory risk factors

**DOI:** 10.3389/fnut.2025.1661468

**Published:** 2025-10-10

**Authors:** Jae B. Park, Renee Peters

**Affiliations:** Diet, Genomics, and Immunology Laboratory, BHNRC, ARS, USDA, Beltsville, MD, United States

**Keywords:** *in vivo* effects of javamide-I/-II, ALT and AST, sE-selectin, sICAM, IGF-1, Growth Hormone, TNF-alpha, MCP-1

## Abstract

**Background:**

Javamide-I/-II (J1/J2) are bioactive compounds found in coffee. Recent studies suggest that J1/J2 may possess anti-inflammatory activity. However, there is no information about the effects of J1/J2 on inflammatory cytokines and other health-related factors *in vivo*.

**Methods:**

To investigate the effects of J1/J2 on inflammatory and other health-related factors (metabolic/growth/hepatic/cardiovascular/ risk factors) *in vivo*, rats were placed into two groups: CG group (a control diet/drinking water, *n* = 10) and JG group (a normal diet/drinking water containing J1/J2, *n* = 10). The study was performed for 16 weeks. During the study, bodyweight and water consumption were monitored weekly, and O-red/HE stains, ALT, AST, IGF1, IGF-1, growth hormone, sE-selectin, sICAM, TNF-alpha, and MCP-1 assays were performed using histochemistry and ELISA methods. The amounts of J1/J2 were measured by HPLC.

**Results:**

The average daily intakes of J1 and J2 were found to be about 0.13 and 0.38 mg, respectively, and no significant difference in bodyweight was found between the CG and JG groups. Also, O-red/HE stains showed no significant difference between both groups, suggesting that J1/J2 may have no adverse effect on the liver. Also, there was no difference in ALT level between both groups. However, the level of AST was significantly lower in the JG group compared to the CG group (*p* < 0.05). Additionally, J1/J2 had no significant effects on growth hormone (GH) and IGF-1 in the JG group, compared to the CG group. Also, there was no significant difference in sE-selectin and sICAM levels between both groups. However, TNF-alpha and MCP-1 levels were significantly lower in the JG group, compared to the CG group (*p* < 0.05), suggesting that J1/J2 may have positive effects on these inflammatory cytokines *in vivo*.

**Conclusion:**

J1/J2 may have beneficial effects on hepatic and inflammatory factors (AST, TNF-alpha and MCP-1) without adverse effects on bodyweight, liver, ALT, GH, IGF-1, sE-selectin, and sICAM in rats.

## Introduction

J1/J2 are bioactive phenolic-conjugated tryptophan compounds found in coffee (1, 2). Regarding health-related effects of J1/J2, several studies indicated that J1/J2 may contain several important biological activities related to human health (3–6). Especially, our studies showed that J1/J2 and their derivatives may possess significant anti-inflammatory activity, particularly anti-cytokine activity *in vitro* (4–8). In line with this, two recent reports even suggested that coffee containing J1/J2 may have some positive effects on inflammatory cytokines (9, 10). However, there is still no information about whether J1/J2 themselves can exert inhibitory effects on inflammatory cytokines *in vivo*. Furthermore, there is no information about potential effects of J1/J2 on other important health-related risk factors such as metabolic, hepatic and cardiovascular risk factors *in vivo*.

Therefore, in this study, potential effects of J1/J2 on metabolic, hepatic, growth, cardiovascular and inflammatory factors (e.g., bodyweight, liver, ALT, AST, GH, IGF-1, sE-selectin, sICAM, TNF-alpha and MCP-1) were investigated in a rodent model. First, the potential effects of J1/J2 on bodyweight were studied, because bodyweight is one of the most reliable metabolic factors for people with not only normal but also over weights. Then, the effect of J1/J2 on the liver was investigated to determine whether J1/J2 can yield any adverse effect on the liver such as inducing abnormal fat accumulation. Also, the levels of two liver enzymes (ALT and AST) were examined to gauge potential effects of J1/J2 on the liver because their levels are reported to increase in several clinical conditions including liver damage, alcoholic/nonalcoholic fatty liver and hepatitis (11, 12). In addition, potential effects of J1/J2 on GH and IGF-1 were investigated, because these two hormones play significant roles in growth, development, metabolism, and other disease conditions such as fatty liver and diabetes (13, 14). Furthermore, the potential effects of J1/J2 on sE-selectin and sICAM were investigated, because these cardiovascular risk factors are reliable biomarkers used for evaluating cardiovascular and other diseases (15–18). Lastly, the potential effects of J1/J2 on TNF-alpha and MCP-1 inflammatory cytokines were investigated, because they are deeply involved in the pathogenesis of numerous disease conditions such as neuroinflammatory diseases, rheumatoid arthritis, cardiovascular diseases, even cancer (19–22). To the best of our knowledge, it is the first report about the effects of J1/J2 on bodyweight, liver, ALT, AST, GH, IGF-1, sE-Selectin, sICAM, TNF-alpha and MCP-1 *in vivo*, providing the first insight to potential health effects of J1/J2 on metabolic, hepatic, cardiovascular and inflammatory risk factors.

## Materials and methods

### Materials

**Figure 1:**
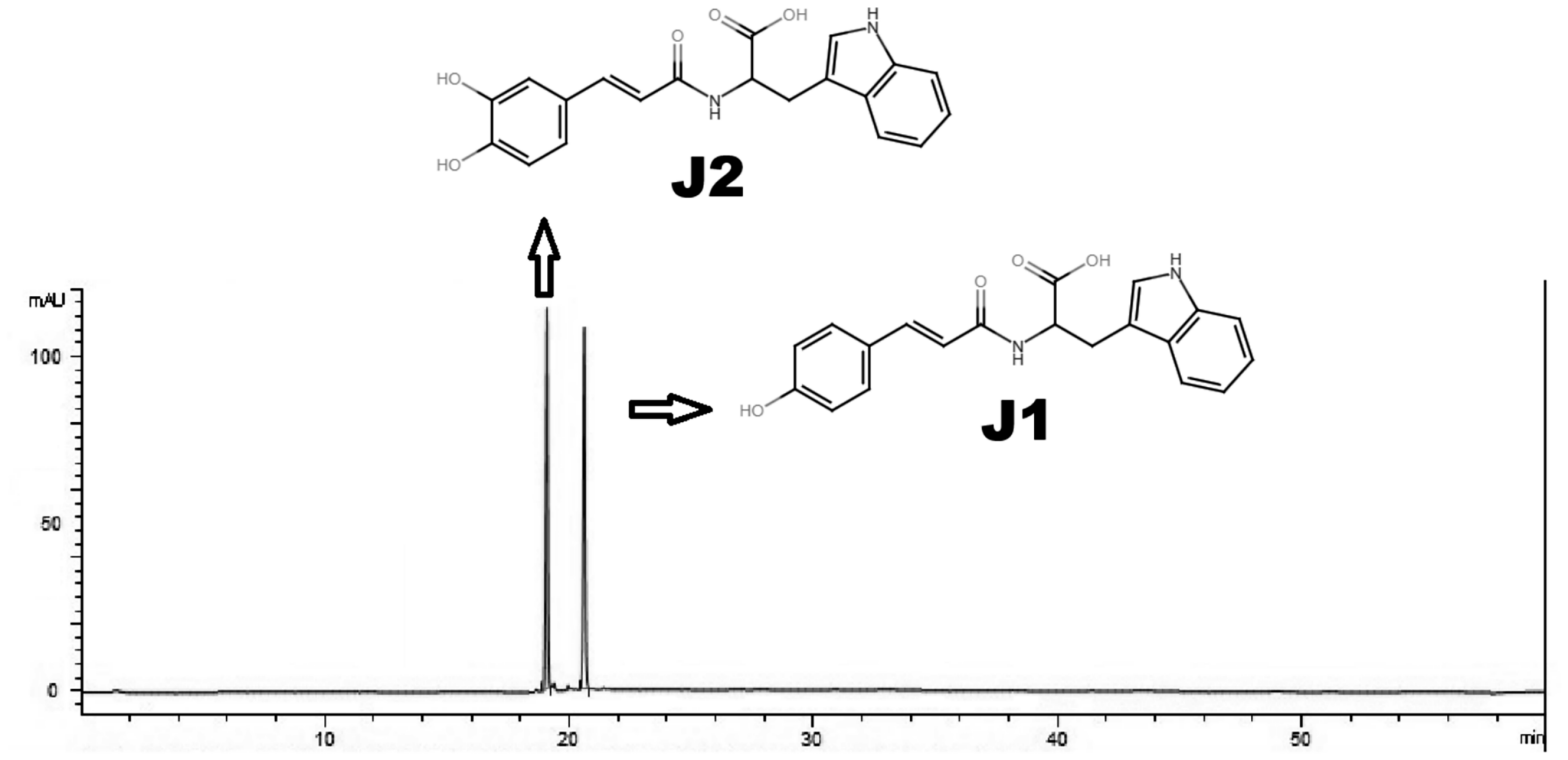
HPLC analysis of J1/J2. J1/J2 were analyzed using HPLC as described in “Materials and Methods.” The peaks are from J1/J2 (100 μM).

Tryptophan, coumaric acid, caffeic acid, methanol, acetonitrile, and other chemicals were purchased from Sigma Chemical Co. (St. Louis, MO). J1/J2 (javamide-I/-II) were synthesized and prepared with more than 98% purity as described previously (Figure 1) (1, 3–5).

### Methods

#### HPLC assay of J1/J2

J1/J2 were analyzed by HPLC as reported previously (1, 8). Briefly, Nova-Pak C18 (Waters, Milford, MA, USA; 150 mm x 2.1 mm i.d., 4 μm) and a two-phase linear gradient condition were used to analyze J1 and J2; buffer A (20 mM NaH_2_PO_4_, pH 4.3) for 0–2 min, a first linear change from buffer A to buffer B (40% acetonitrile) for 2–18 min, a second linear change from buffer B (40% acetonitrile) to buffer B (60% acetonitrile) for 18–25 min and buffer B (60% acetonitrile) for 5 min at the flow rate of 1 mL/min. J1 and J2 were prepared in buffer A (20 mM NaH_2_PO_4_, pH 4.3) and the samples (10 μL) were injected by an autosampler into the Agilent 1,260 Infinity Quaternary LC system (Agilent technologies, Santa Clara, CA, USA) and were monitored by a photo diode array detector (1,260 DAD) (Agilent technologies, Santa Clara, CA, USA). This HPLC method with a limit of detection (3 μg/mL), and a limit of quantitation (4 μg/mL) can separate J1 and J2 with distinctive retention times (Figure 1) as reported previously (8).

#### Preparation of J1/J2 containing water for animal study

Based on several studies, the amounts of J1/J2 were found to be ≤ 1 and ≤ 3 mg in a cup of coffee (250 mL), respectively (2, 8, 9). Therefore, for this study, coffee (250 mL) was prepared with J1 (1 mg) and J2 (3 mg). Usually, three cups per day are the daily average for many coffee drinkers. Thus, total amounts of J1/J2 per day are as follows: J1 (3 mg) and J2 (9 mg). Using the FDA guideline “Estimating the Maximum Safe Starting Dose in Initial Clinical Trials for Therapeutics in Adult Healthy Volunteers,” J1 (0.13 mg/0.5 kg/day) and J2 (0.40 mg/0.5 kg/day) were prepared in 30 mL of reverse osmosis water, as reported in our previous study (9).

#### Animal study

Eight-week Sprague–Dawley male rats (n = 20) were purchased from Charles River (Wilmington, MA) and housed in ventilated micro-isolator racks with an automatic watering system in a room with a 12:12-h light/dark cycle and ambient temperature of 18–23 °C with relative humidity of 55.5%. The animals were acclimated to experimental conditions for 2 weeks. Then, the animal study was conducted according to an animal protocol approved by the Beltsville Area Animal Care and Use Committee (AUP Approval No. 13–025) which was in accordance with the Institutional Animal Care and Use Committee (IACUC). Also, this study was performed in accordance with relevant guidelines and regulations, and all methods are reported in accordance with ARRIVE guidelines. Rats were fed an AIN-76A purified diet^1^ and provided with reverse osmosis water to provide the recommended allowance of all nutrients and water required for optimal health. For the study, rats were assigned into two groups; water control group (CG; *n* = 10) and J1/J2 group (JG; *n* = 10), because the preliminary data showed that 10 rats in each group often provide a power of higher than 0.99 to detect 25% difference in means with a standard deviation of 10%. For the JG group, J1/J2 containing water was prepared for the study described above. During the study (16 weeks), J1/J2 containing water was provided to the JG group, meanwhile reverse osmosis water was provided to the CG group. Also, food and water were provided to rats *ad libitum* during the study. However, water consumption was monitored weekly to determine the daily intakes of J1/J2. Also, the bodyweight of rats in both groups was monitored weekly. During this study, no anesthesia method was used. At the end of the experiment, after 16 h fasting, rats (*n* = 20) were individually euthanized by CO_2_ euthanasia, which is commonly used for euthanasia of small rodents. After checking the absence of vital signs (i.e., heartbeat, respiration, and response to painful stimuli), blood was immediately collected into EDTA-coated vials and centrifuged (3,000 rpm for 10 min) for the plasma. All plasma samples were stored at -80 °C until analyzed. Also, the livers were surgically removed from animals and liver tissue sections were fixed with formalin (10%).

#### Oil red O and HE stains

Oil Red O staining was performed as described previously (23). Briefly, liver tissue sections were fixed with formalin (10%) as described above. For Oil Red O staining, formalin was discarded, and the sections were washed twice using deionized water. Then, 100% propylene glycol was added to the sections for 5 min. After that, the sections were coated evenly with heated oil red O solution for 12 min. After that, the oil red O solution was discarded, and the sections were washed 2–5 times with water. Then, the Mayer’s hematoxylin solution was added to the sections and incubated for 10 s. The hematoxylin solution was discarded, and the sections were washed 2–5 times with water. The slides were covered with glycerin jelly mounting medium and examined using PANNORAMIC 1000 (3D Histech Ltd., Budapest, Hungary). Additionally, HE (hematoxylin/eosin) stain was performed as described previously (24). The fixed tissue sections were placed in Mayer’s hematoxylin for 3 min. After that, the slides were placed in a bluing solution for 1 min and 40 s. The slides were placed in 100% alcohol for 5 min, then placed in alcohol-based Eosin for 1 min. Slides were placed in xylene for clearing for 2 min, then undergo 2 changes of 100% alcohol dehydration for 1 min each. Again, slides were placed in xylene for clearing for 2 min. Coverslips were mounted on the slides using a xylene based mounting medium, and the samples were scanned using PANNORAMIC 1000 (3D Histech Ltd., Budapest, Hungary).

#### ALT and AST analyses

The levels of alanine aminotransferase (ALT) and aspartate aminotransferase (AST) in plasma samples were measured using commercial ALT (Cat # ab285264) and AST (Cat # ab263883) kits (Abcam, Cambridge, MA, the United States) according to the manufacturer’s instructions.

#### Measurements of GH and IGF-1

GH level was measured in plasma samples using a commercial kit (Rat/Mouse Growth Hormone ELISA, Cat #EZRMGH-45 K, Millipore, Billerica, MA, the United States) according to the manufacturer’s instructions. The hormone level was measured spectrophotometrically by the increased absorbency at 450 nm, which is directly proportional to the amount of rat/mouse growth hormone in the samples. IGF-I level was also measured in plasma samples using a commercial kit (Rat/Mouse IGF-I Quantikine ELISA Kit (Cat # MG100), R&D systems, Minneapolis, MN, the United States), which was measured spectrophotometrically by the increased absorbency at 450 nm, according to the manufacturer’s instructions.

#### Measurements of sICAM and sE-selectin

Rat soluble intercellular adhesion molecule 1 (sICAM) level was determined in plasma samples using sICAM ELISA kit (Cat # RIC100, R&D systems, Minneapolis, MN, the United States) according to the manufacturer’s instructions. The level of sICAM was measured spectrophotometrically by the increased absorbency at 450 nm, directly proportional to the amount of sICAM in the samples. sE-selectin level was determined in the plasma samples using rat sE-Selectin ELISA Kit (Cat. # ab171334; Abcam, Cambridge, MA, the United States) according to the manufacturer’s protocols. The level of sE-Selectin was measured spectrophotometrically by the increased absorbency at 450 nm.

#### Measurements of TNF-alpha and MCP-1

The levels of TNF-alpha and MCP-1 were measured in plasma samples using TNF-alpha (Cat. # KRC3011; Thermos Fisher, Camarillo, CA, the United States) and MCP-1 Quantikine ELISA kits (Cat. # DY3144-05; R&D systems, Minneapolis, MN, the United States) according to the manufacturer’s protocols. Briefly, assay was performed using 100 μL of plasma sample with background and standard controls at room temperature. Both kits detect endogenous and recombinant rat TNF-alpha and MCP-1 without low cross-reactivity against other related proteins.

### Statistical analysis

Statistical analyses were performed with Sigma Plot 11.0 (Chicago, IL). *p-*value was calculated using the t-test method, and *p* < 0.05 was considered as statistically significant. Also, the size effects (d) were calculated using the Cohen’s d value. Data points were represented as the mean ± SD (*n* = 10).

## Results

### Preparation of J1/J2 in water and HPLC analysis

The amounts of J1/J2 used for animal study were analyzed using HPLC as described in “Materials and Methods” (Figure 1). Based on our previous studies, the amounts of J1 and J2 were found to be ≤ 1 and ≤ 3 mg per cup of coffee (250 mL), respectively. Based on this, the total amounts of J1/J2 in three cups are as follows: J1 (3 mg) and J2 (9.0 mg). Using the FDA guideline “Estimating the Maximum Safe Starting Dose in Initial Clinical Trials for Therapeutics in Adult Healthy Volunteers,” J1/J2-containing water for the study was prepared by adding J1 (0.13 mg) and J2 (0.40 mg) in water (30 mL) as described in “Materials and Methods” (9).

### Determination of J1/J2 intake

**Figure 2:**
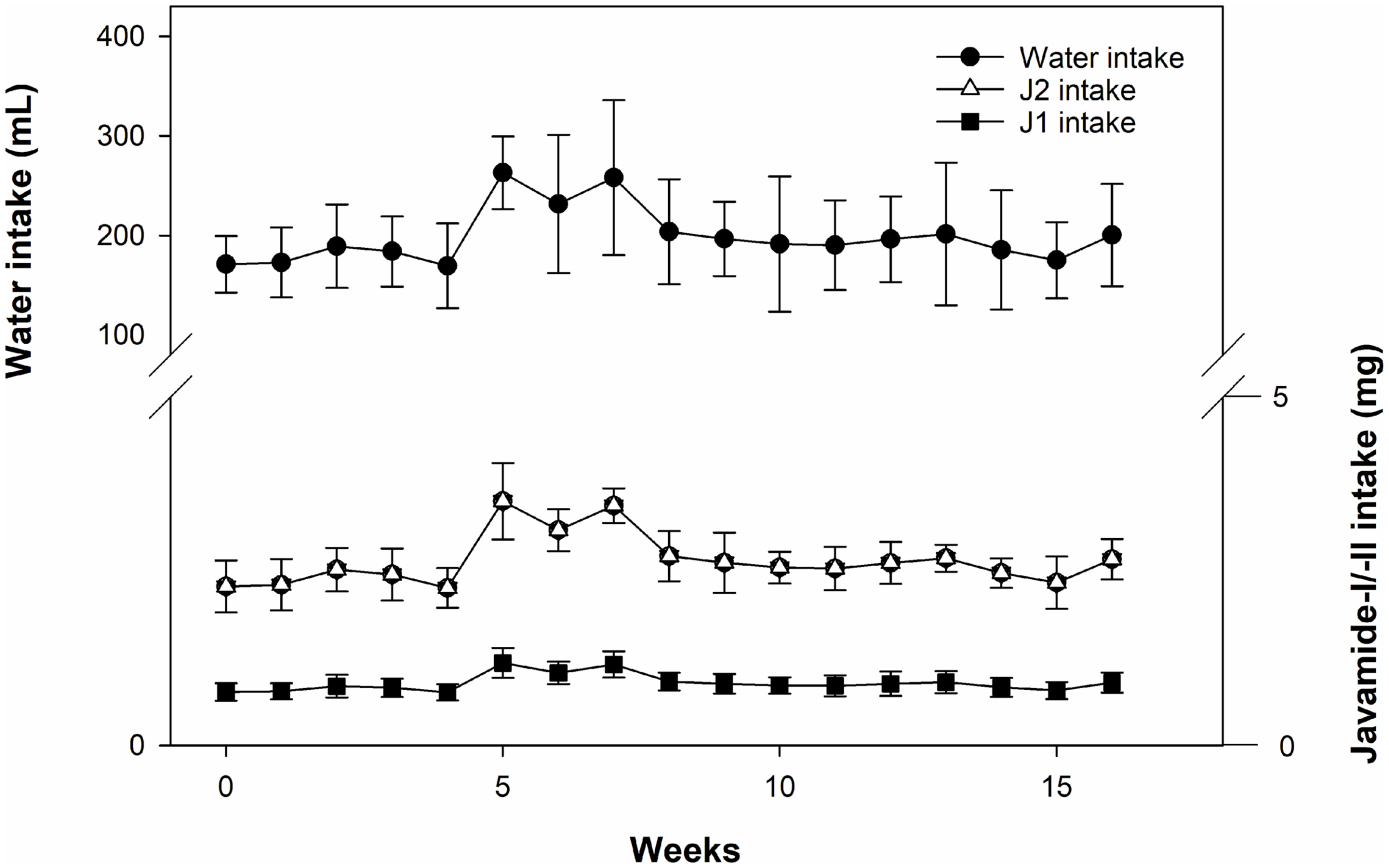
Water consumption and J1/J2 intakes. The intakes of J1/J2 were calculated based on water consumption in the JG group. Data is presented as the mean ± SD (*n* = 10).

Using the data of weekly water consumption, J1 and J2 intakes were calculated (Figure 2). The weekly average intakes of J1 and J2 were found to be about 0.9 and 2.65 mg, so the daily average intakes were about 0.13 and 0.38 mg. These numbers were compatible with the daily amounts of J1 and J2 (0.13 and 0.4 mg) calculated using the FDA guideline.

### Effects of J1/J2 on bodyweight

**Figure 3:**
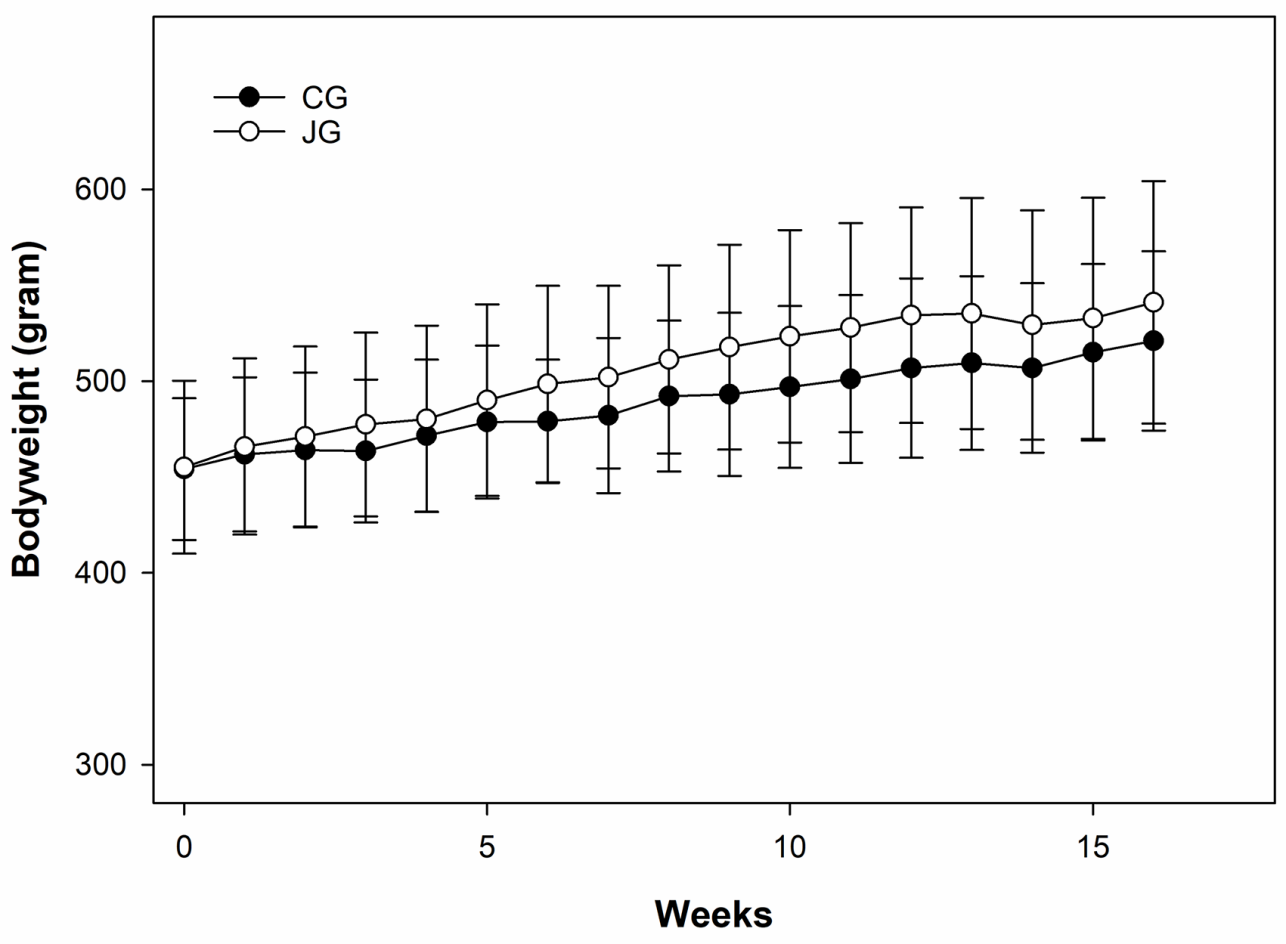
Effects of J1/J2 on bodyweight. There was no significant difference in bodyweight between the CG and JG groups. Data was analyzed using the t-test method. Bodyweight and chow consumption were not significantly different between the groups. Data is presented as the mean ± SD (*n* = 10).

Like water consumption, bodyweight was also monitored weekly during the study. As shown in Figure 3, there was a very similar pattern of bodyweight gain between the groups, and no significant difference was found in bodyweight between the groups during the study. As expected, no difference in bodyweight was found between the groups at the end of the study. This data suggests that J1/J2 may not have significant effects on bodyweight in the JG group, compared to the CG group.

### Effects of J1/J2 on the liver

**Figure 4:**
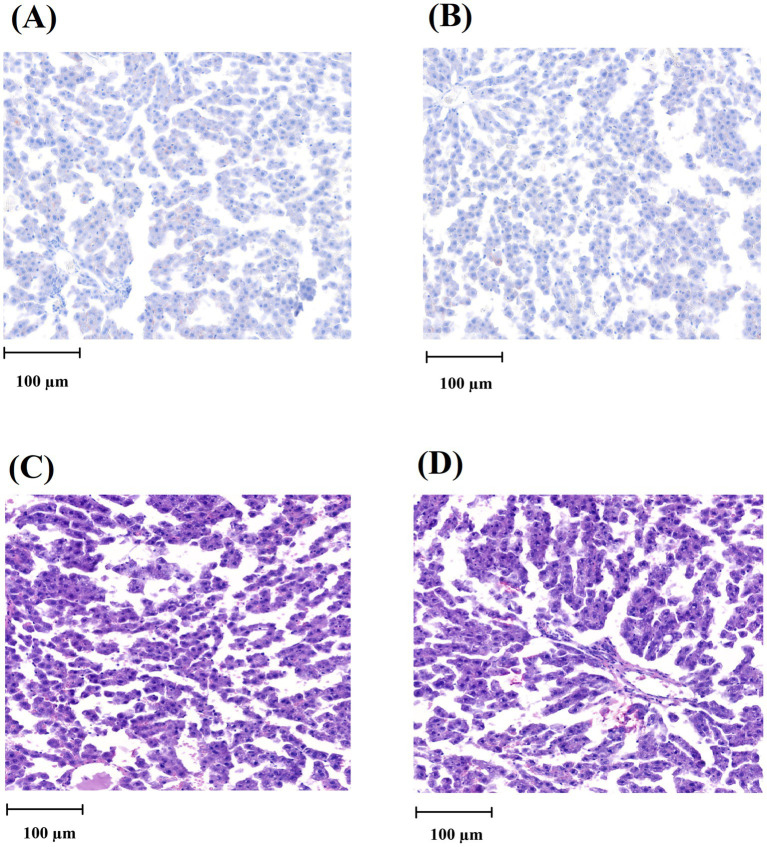
Histological analysis of the liver tissue. **(A)** Oil Red O staining image of the liver from the CG group. **(B)** Oil Red O staining image of the liver from the JG group. **(C)** HE-stained image of the liver from the CG group. **(D)** HE-stained image of the liver from the JG group. The scale of the bar (100 μm) is detonated in each picture.

The liver is one of the organs able to react to harmful chemicals via initiating/developing fatty liver and other liver diseases (25, 26). Therefore, the effect of J1/J2 on the liver was investigated by Oil Red O staining, because this is one of the most used methods in histology and pathology to detect/visualize neutral lipids and triglycerides in frozen sections of tissue samples including the liver samples (23, 24). As shown in Figure 4, both liver samples did not produce a vivid red color often seen in the fatty livers, suggesting that there was no significant lipid accumulation in the liver samples from both groups. Additionally, the hematoxylin/eosin (HE) staining was performed, because the hematoxylin staining is commonly used to identify tissue structures, cell types and morphological changes via showing cell nuclei in a purplish-blue color and staining extracellular matrix and cytoplasm with a pink color (23, 24). Likewise, there was no significant difference in the structural patterns of cell nuclei and cytoplasm in the liver samples from the CG and JG groups, suggesting that there may be no significant pathological changes in the livers from the JG group, compared to those from the CG group (Figure 4). This data suggests that J1/J2 may have no adverse effect on the liver in the JG group, compared to the CG group.

### Effects of J1/J2 on ALT and AST

**Figure 5:**
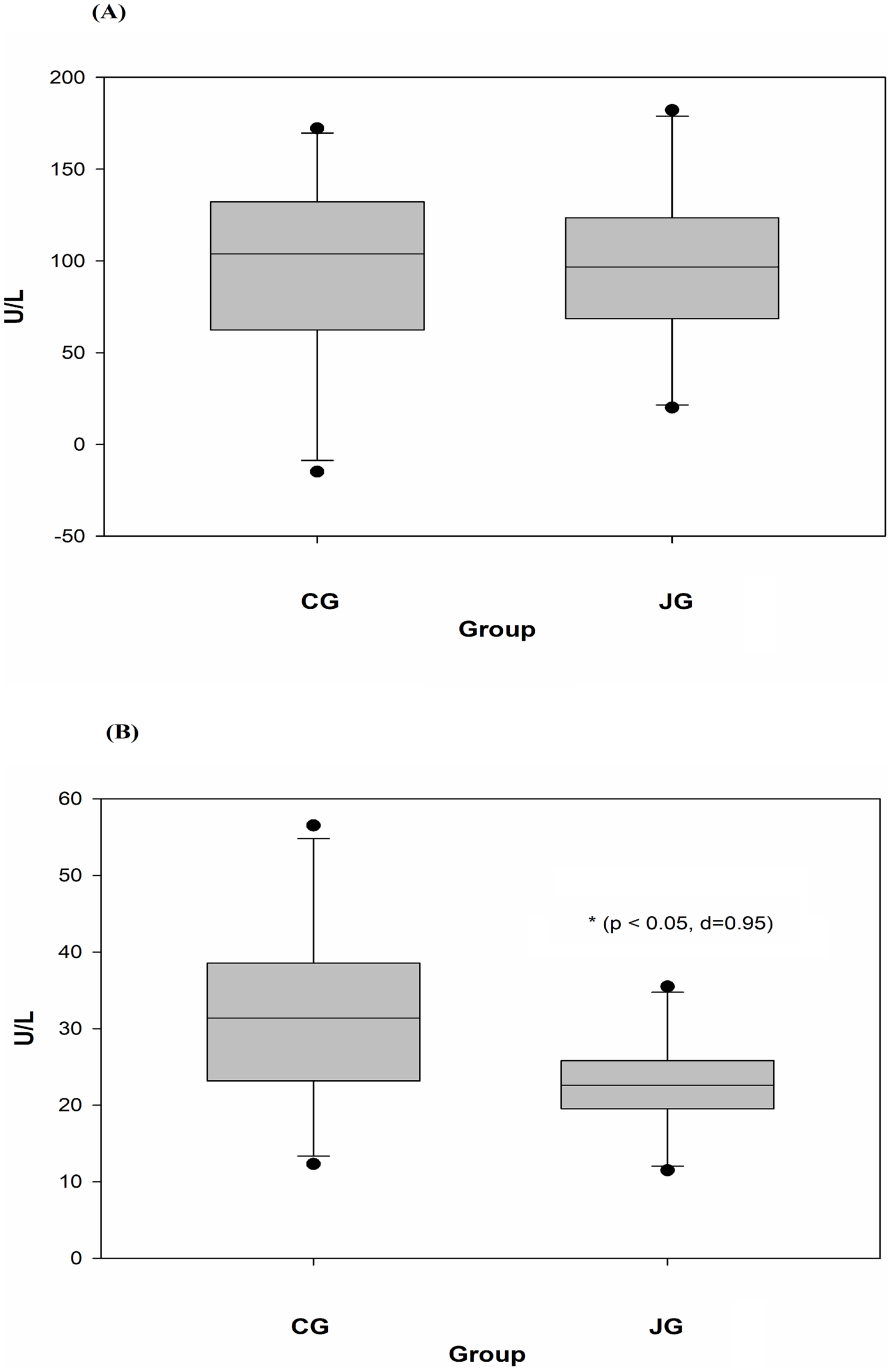
Effects of J1/J2 on ALT and AST. ALT **(A)** and AST **(B)** were measured using plasma samples. There was no significant difference in ALT level between the CG and JG groups, but the level of AST was lower in the JG groups, compared to the CG group. Data was analyzed using the *t*-test method and data is presented as the mean ± SD (*n* = 10) with *p*-value and *d* (Cohen’s *d*) value. The mark (*) indicates statistical significance compared to the CG group (*p* < 0.05).

Potential effects of J1/J2 on the liver were further studied by measuring ALT (alanine aminotransferase) and AST (aspartate aminotransferase) levels in the samples, because high levels of ALT and AST are often detected in the blood from individuals with several clinical conditions such as liver damage, alcoholic/nonalcoholic liver injury and hepatitis (11, 12). The levels of ALT and AST were measured in plasma samples as described in “Materials and Methods.” As shown in Figure 5A, there was no statistically significant difference in ALT level between the CG and JG groups. Though, the average level of ALT was found to be lower in the JG group compared to the CC group, suggesting J1/J2 may have some potential effects on the liver. However, the level of AST was found to be lower significantly in the JG group, compared to the CG group (*p* < 0.05) (Figure 5B), suggesting J1/J2 may have beneficial effects on AST.

### Effects of J1/J2 on GH and IGF-1

**Figure 6:**
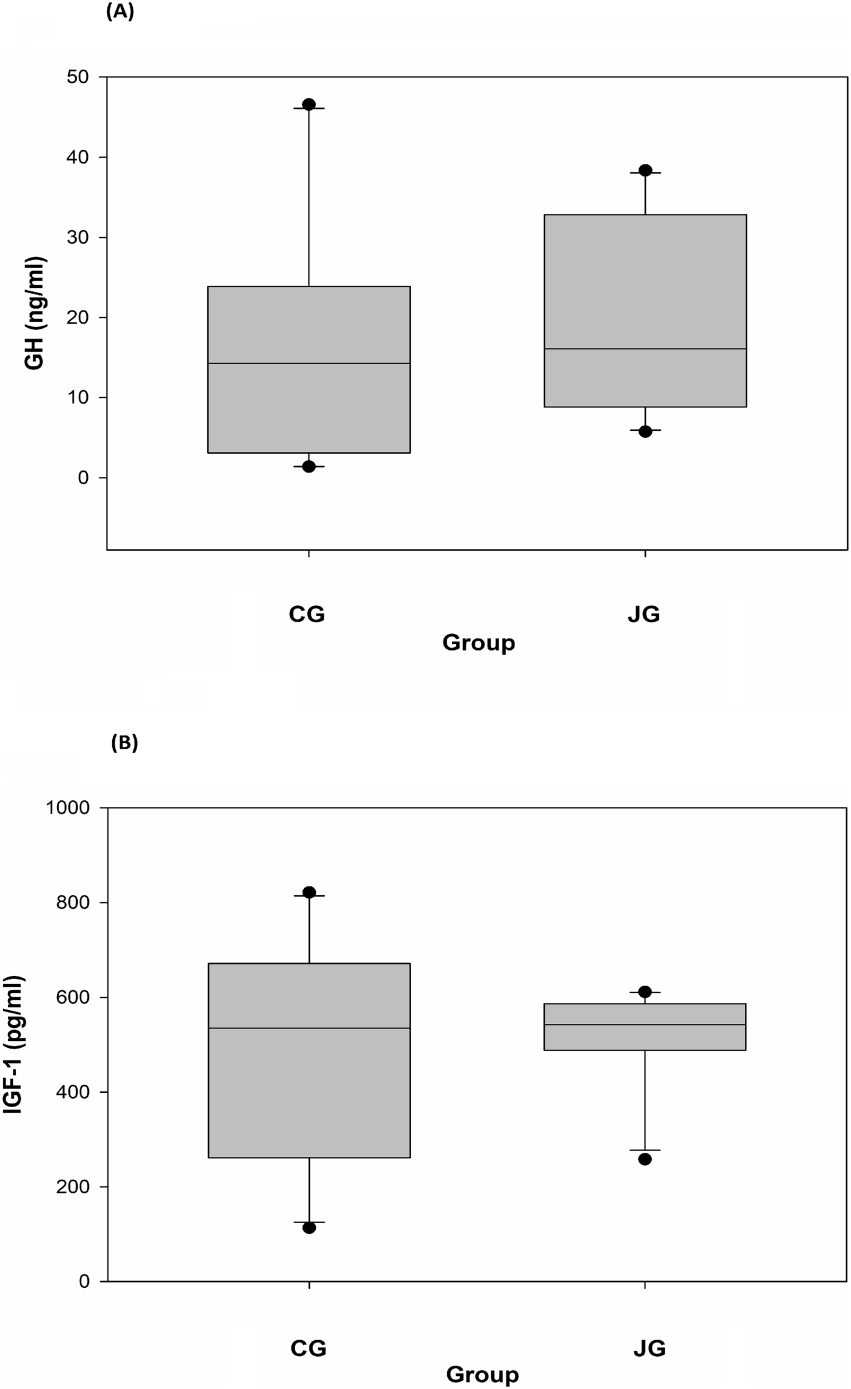
Effects of J1/J2 on GH and IGF-1. GH **(A)** and IGF-1 **(B)** were measured using plasma samples. There were no significant differences in GH and IGF-1 levels between the CG and JG groups. Data is presented as the mean ± SD (*n* = 10).

The effect of J1/J2 on GH was investigated in plasma samples, and the data showed that there was no significant difference in plasma level of GH between the CG and JG groups. In fact, the average level of GH was found to be a bit higher in the JG group than the CG group (Figure 6A), suggesting that J1/J2 may have no negative effect on the hormone. Also, the effect of J1/J2 on IGF-1 was investigated in plasma samples, because IGF-1 plays a crucial role in growth and development, and because IGF-1 is produced primarily by the liver in response to growth hormone stimulation (12, 13). As shown in Figure 6B, there was no significant difference in plasma IGF-1 level between both groups, suggesting that J1/J2 may have no adverse effects on GH and IGF-1.

### Effects of J1/J2 on sICAM-1 and sE-selectin

**Figure 7:**
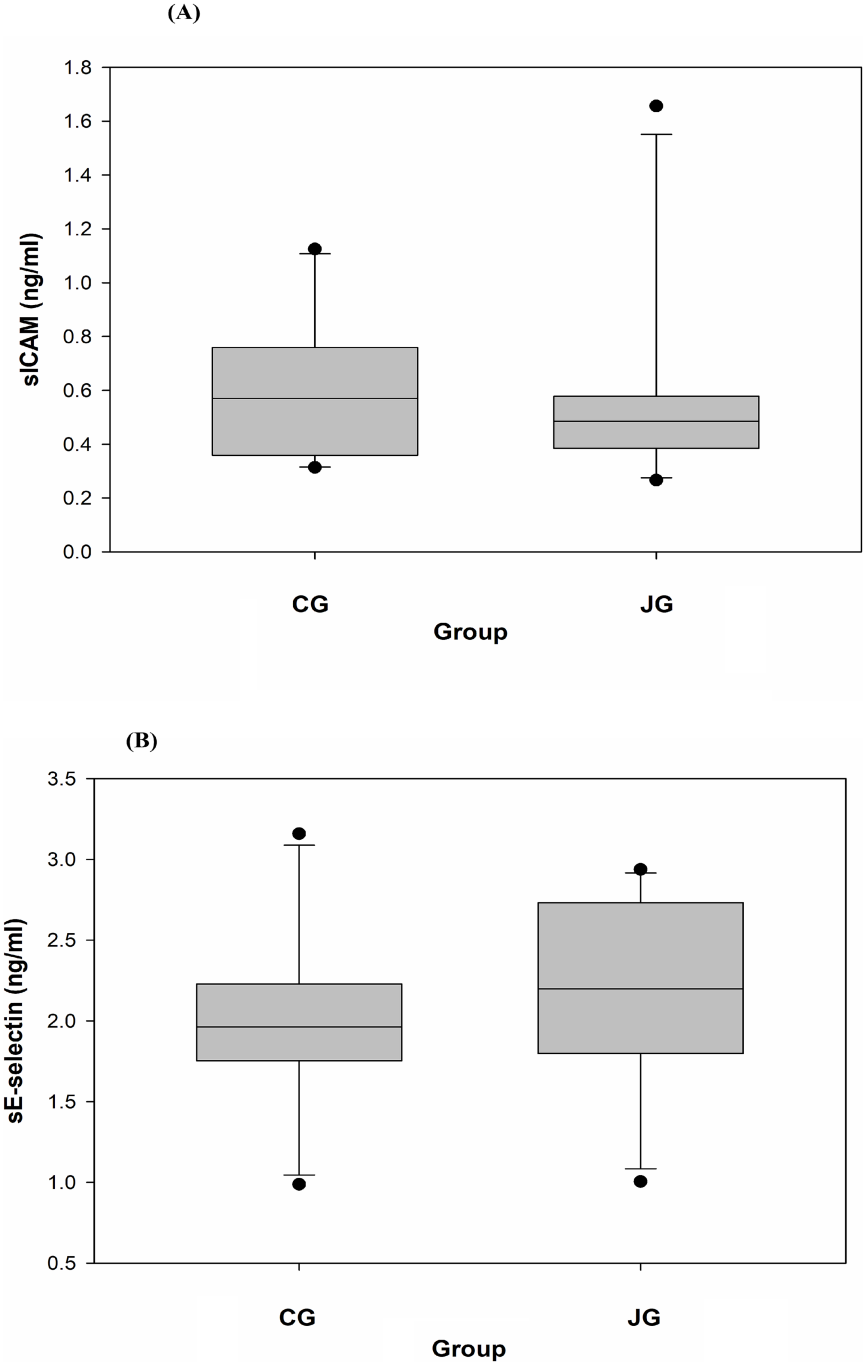
Effects of J1/J2 on sICAM and sE-selectin. sICAM **(A)** and sE-selectin **(B)** were measured using plasma samples. There were no significant differences in sICAM and sE-selectin levels between the CG and JG groups. Data is presented as the mean ± SD (*n* = 10).

The effect of J1/J2 on sICAM-1 was investigated because sICAM-1 is commonly used as an atherosclerotic risk factor in patients with progressed CVD (15, 16). The data showed that there was no significant difference in plasma sICAM-1 levels between the CG and JG groups, although the average level of sICAM was lower in the JG group than the CG group (Figure 7A). Next, the effect of J1/J2 on sE-selectin expression was investigated in plasma samples, because sE-selectin is also a cardiovascular/inflammatory biomarker (17, 18). Again, the data showed that there was no significant difference in plasma sE-selectin levels between both groups (Figure 7B). This data suggests that J1/J2 may have no negative effect on plasma sICAM-1 and sE-selectin levels in the JG group, compared to the CG group.

### Effects of J1/J2 on TNF-alpha and MCP-1

**Figure 8:**
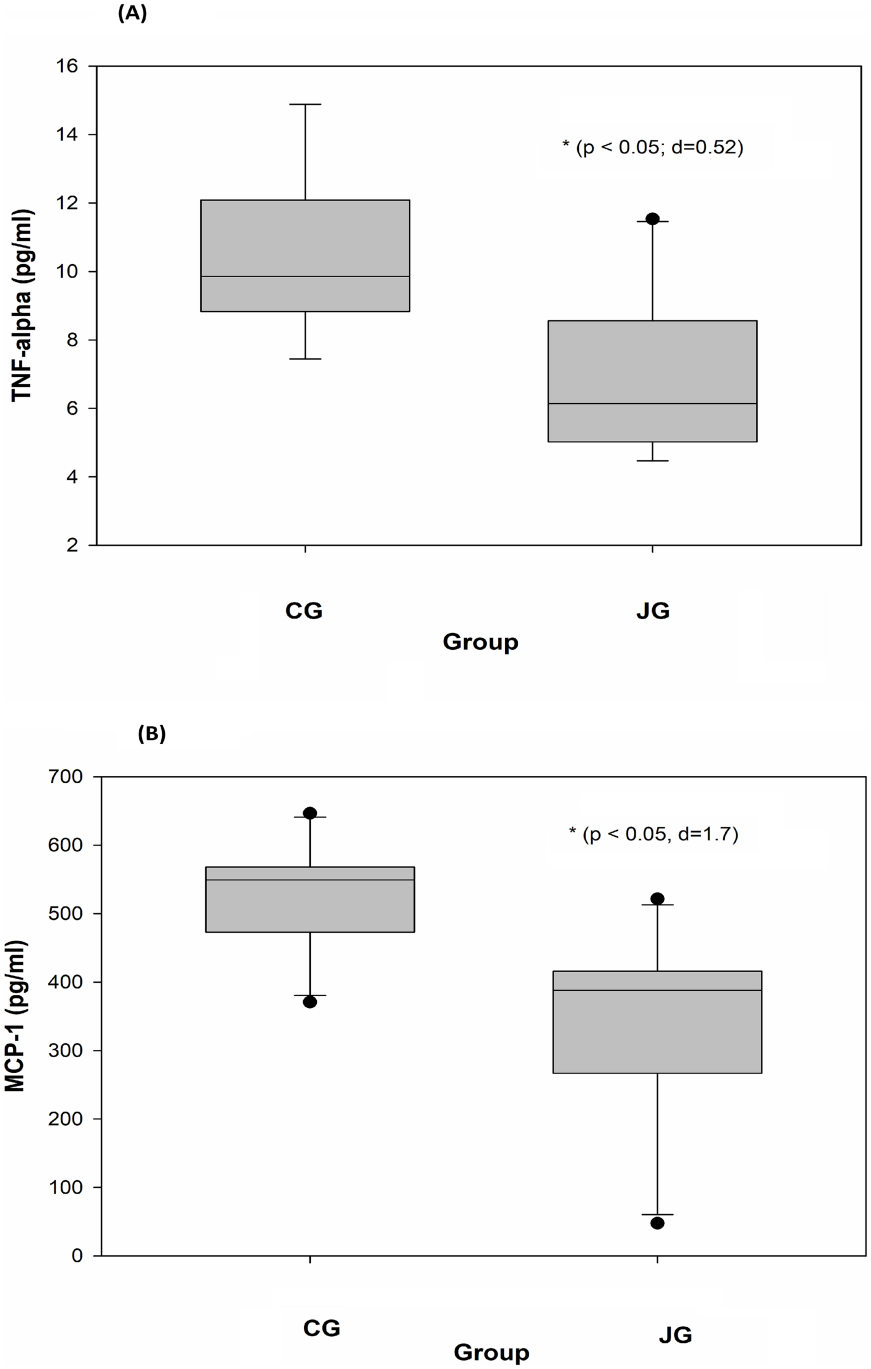
Effects of J1/J2 on TNF-alpha and MCP-1. TNF-alpha **(A)** and MCP-1 **(B)** were measured using plasma samples. There were significant differences in TNF-alpha and MCP-1 levels between the CG and JG groups. Data was analyzed using the *t*-test method. Data is presented as the mean ± SD (*n* = 10) with *p*-value and *d* (Cohen’s *d*) value. The mark (*) indicates statistical significance compared to the CG group (*p* < 0.05).

Tumor necrosis factor-alpha (TNF-alpha) is a multifunctional cytokine. For instance, in the liver, TNF-alpha causes numerous biological responses including liver inflammation (19, 20). Like TNF-alpha, MCP-1 is also associated with the pathogenesis of numerous disease conditions such as metabolic, neuroinflammatory diseases, rheumatoid arthritis and cardiovascular diseases (21, 22). Interestingly, several reports suggest that J1/J2 may have positive effects on inflammatory cytokines *in vitro* (4–7). However, there is currently no information about the effects of J1/J2 on inflammatory cytokines *in vivo*. Therefore, the potential effects of J1/J2 on TNF-alpha and MCP-1 were investigated in plasma samples. As shown in Figure 8A, there was a significant difference in plasma level of TNF-alpha between the CG and JG groups; the level of TNF-alpha was significantly lower in the JG groups than the CG group (*p* < 0.05). This data suggests that J1/J2 may have positive effects on TNF-alpha. Next, the potential effect of J1/J2 on MCP-1 was investigated in the samples. As shown in Figures 8B, a significant difference was also found in plasma MCP-1 level between the CG and JG groups, and the level of MCP-1 was significantly lower in the JG groups than the CG group (p < 0.05), suggesting that J1/J2 may have positive effects on MCP-1. Based on the data, J1/J2 is likely to have some beneficial effects on both inflammatory cytokines (TNF-alpha and MCP-1) in the JG group, compared to the CG group (*p* < 0.05). Altogether, the data suggests that J1/J2 may have beneficial effects on hepatic and inflammatory factors (AST, TNF-alpha and MCP-1) without adverse effects on ALT, sE-selectin, sICAM, IGF-1 and GH *in vivo* (Table 1).

**Table 1:** Summary of J1/2 effects on metabolic, hepatic, cardiovascular and inflammatory factors.

Factors	CG group	JG group	*p*-values
Bodyweight	521.0 ± 46.7	541.1 ± 63.2	*p* = 0.429
ALT	95.3 ± 53.3	96.5 ± 47.6	*p* = 0.938
AST	63.5 ± 23.5	45.2 ± 5.6	*p* = 0.021*
GH	16.9 ± 15.6	19.9 ± 11.8	*p* = 0.571
IGF-1	499.2 ± 228.9	517.9 ± 102.7	*p* = 0.970
sICAM	0.60 ± 0.27	0.57 ± 0.39	*p* = 0.240
sE-selectin	2.0 ± 0.66	2.2 ± 0.58	*p* = 0.451
TNF-alpha	10.5 ± 2.6	6.9 ± 2.5	*p* = 0.015*
MCP-1	528.8 ± 76.3	338.7 ± 136.9	*p* = 0.001*

Data were represented as the mean ± SD (*n* = 10). *p-*value was calculated using the *t*-test method, and the mark (*) indicates statistical significance compared to the CG group (*p* < 0.05). AST, aspartate aminotransferase; GH, growth hormone; IGF-1, insulin-like growth factor 1; sE-selectin, soluble E-selectin; sICAM, soluble intercellular adhesion molecule 1; TNF-alpha, tumor necrosis factor-alpha; MCP-1, monocyte chemoattractant protein-1.

## Discussion

J1/J2 are bioactive phenolic-conjugated tryptophan compounds found in coffee beans and products (1, 2). Previous studies showed that many coffee beans/products in the market may contain J1/J2 (1, 2, 9, 10). Thus, people are likely to intake J1 and J2 together when they drink coffee. Additionally, recent *in vitro* studies suggest that both J1 and J2 may contain several health-related activities including anti-inflammatory activity (3–7). However, there is no information about anti-inflammatory activity of J1/J2 *in vivo*. Furthermore, the effects of J1/J2 on other important health factors (bodyweight, liver, ALT, AST, GH, IGF-1, sE-selectin, sICAM) have not been investigated at all. Therefore, in this study, potential effects of J1/J2 were investigated related to these risk factors *in vivo*. Particularly, in this study, the FDA guideline “Estimating the Maximum Safe Starting Dose in Initial Clinical Trials for Therapeutics in Adult Healthy Volunteers” was used to prepare the drinking water containing J1/J2, and the daily intakes of J1/J2 were calculated. In fact, this type of data is often missing in many *in vivo* studies, being difficult to correlate study outcomes to the doses of tested compounds.

As shown in Figure 3, both groups showed a very similar pattern of weight gains during the study, suggesting that J1/J2 may have no adverse impact on bodyweight. Next, potential effects of J1/J2 on liver were investigated, because harmful/toxic chemicals have been alleged to initiate/develop fatty liver disease (25–27). For example, nonalcoholic fatty liver disease (NAFLD) has been reported to ensue with exposure to drugs, alcohol, pesticides and pollutants (25–27). Therefore, the effects of J1/J2 were investigated related to fatty livers. As shown in Figure 4, there was no significant difference in the color of Oil Red-O staining between the CG and JG groups, suggesting that J1/J2 may not induce fatty liver in the JG group, compared to the CG group. Additionally, we investigated the effect of J1/J2 on the liver using two liver-related risk factors (e.g., ALT, AST). As shown in Figure 5A, plasma ALT level was not significantly different between both groups. However, the data showed that the average level of ALT was lower in the JG group than the CG group, suggesting that J1/J2 may have some potential in lowering ALT level. Like ALT, AST is also a good biomarker for assessing the progression of fatty liver. In fact, AST is often viewed as a broader risk factor than ALT, because AST is produced from not only the liver but also other organs (e.g., heart, muscle, kidneys, brain, and blood cells) (11). As shown in Figure 5B, the plasma AST level of the JG group was found to be significantly lower than the CG group, suggesting that J1/J2 may have some beneficial effects on not only the liver but also other organs such as heart, muscle, and blood cells. In this study, the daily average intakes of J1/J2 were about 0.13 and 0.38 mg during the study (Figure 2). So, these amounts of J1/J2 may be correlated with the beneficial effects on AST in the JG group. Although ALT and AST assessments are commonly considered as useful indicators of liver function/injury, there are other biomarkers for liver function/injury such as alkaline phosphatase, gamma-glutamyl transferase, serum albumin and total protein. However, these biomarkers could not be tested herein due to the scarcity of plasma samples. Therefore, in the future, these may be investigated related to liver function and injury to provide a more complete assessment for the hepatic effects.

Related to coffee consumption, there is an old saying “coffee stunts growth.” Therefore, the effect of J1/J2 on growth hormone was investigated. As shown in Figure 6A, plasma GH levels were not significantly different between both groups. However, the average level of GH was found to be higher in the JG group than the CG group, suggesting that J1/J2 may have no negative effect on GH. Also, there was no significant difference in the plasma IGF-1 level between the JG and CG groups (Figure 6B). Together, J1/J2 may have no negative effects on GH and IGF-1 levels, suggesting that J1/J2 may have no opposing effects on growth, development, anabolism, and metabolism. Next, the effects of J1/J2 on sICAM were investigated, because sICAM is often used as a risk factor for several diseases such as inflammatory condition, infections, autoimmune disease, and cardiovascular diseases (28–30). As shown in Figure 7A, plasma sICAM levels were not significantly different between both groups. However, the average level of sICAM was found to be lower in the JG group than the CG group, suggesting that the high levels of J1/J2 may have some positive effects on sICAM in the JG group, compared to the CG group. Like sICAM, E-selectin is a good biomarker for assessing the progression of cardiovascular diseases such as atherosclerosis, and the soluble form (sE-selectin) can be found in the blood of patients with cardiovascular diseases (31, 32). Therefore, the effect of J1/J2 on sE-selectin was evaluated in plasma samples from both groups. As shown in Figure 7B, there was no significant difference in plasma sE-selectin level between the JG and CG groups. This data suggests that J1/J2 may have no adverse effects on metabolic factors (GH and IGF-1) and cardiovascular risk factors (sICAM and sE-selectin).

Inflammatory cytokines are significantly involved in the development/progress of several human chronic diseases such as diabetes, liver, kidney and cardiovascular diseases (33–36). Among them, TNF-alpha is a pro-inflammatory cytokine being involved in the development and progression of several inflammatory and autoimmune diseases (37, 38). Especially in the liver, TNF-alpha is involved in numerous biological outcomes such as hepatocyte apoptosis and necroptosis, liver inflammation, and hepatocellular carcinoma (38). Therefore, in this study, *in vivo* effects of J1/J2 on TNF-alpha were investigated. As shown in Figure 8A, the level of TNF-alpha was significantly lower in the JG group in comparison to the CG group, suggesting that J1/J2 may provide some positive effects on TNF-alpha level in the JG group, compared to the CG group. Considering the various functions of TNF-alpha in the liver and other parts of the human body, it is likely that J1/J2 may have potential to exert some positive effects on TNF-alpha related diseases. Like TNF-alpha, MCP-1 (Monocyte chemoattractant protein-1) is one of the CC family of chemokines, which plays a critical role in the process of inflammation via attracting or enhancing the expression of other inflammatory factors/cells. Also, MCP-1 is believed to be associated with numerous disease conditions such as infection, inflammation, neuroinflammatory diseases, rheumatoid arthritis, and cardiovascular diseases (35, 36, 39, 40). Especially, plasma levels of MCP-1 are reported to be a risk factor for patients with acute coronary syndromes and other cardiovascular diseases (35, 39). Therefore, the potential effects of J1/J2 on MCP-1 were investigated herein. As shown in Figure 8B, the level of MCP-1 was found to be significantly lower in the JG group, compared to the CG group (Figure 8B). This data indicates that J1/J2 can have some beneficial effects on not only TNF-alpha but also MCP-1 *in vivo*. As mentioned previously, the daily average intakes of J1/J2 were, respectively, about 0.13 and 0.38 mg during the study (Figure 2). Thus, these amounts may be sufficient for exerting positive effects on TNF-alpha and MCP-1 in the rats. In fact, this is the first study about potential health effects of J1/J2, particularly the data about AST, TNF-alpha and MCP-1 is promising. However, the study was conducted using normal animals without inducing any disease condition. Thus, the data is likely about beneficial changes in hepatic (AST) and inflammatory biomarkers (TNF-alpha and MCP-1) under a normal physiological condition. Therefore, in the future, potential effects of J1/J2 should be investigated using an appropriate disease model to evaluate their effects and possible underlying mechanisms. Also, pharmacological/clinical studies should be conducted to investigate the potential effects of J1/J2 on a broad range of human health in the future, because there is no information about the potential health effects of J1/J2 in humans. In summary, the data suggests that J1/J2 may have no adverse effects on bodyweight, liver, ALT, GH, IGF-1, sE-selectin, and sICAM selectin, but they may have some beneficial effects on AST, TNF-alpha, and MCP-1 in the JG group, compared to the CG group.

## Data Availability

The original contributions presented in the study are included in the article/supplementary material, further inquiries can be directed to the corresponding author/s.

## Footnotes

^1^ https://dyets.com/research-diets-directory/ain-76a-purified-rodent-diet/
